# Intravenous metoclopramide for increasing endoscopic mucosal visualization in patients with acute upper gastrointestinal bleeding: a multicenter, randomized, double-blind, controlled trial

**DOI:** 10.1038/s41598-024-57913-2

**Published:** 2024-03-31

**Authors:** Paveeyada Manupeeraphant, Dhanusorn Wanichagool, Thaphat Songlin, Piyarat Thanathanee, Nalerdon Chalermsuksant, Karjpong Techathuvanan, Supatsri Sethasine

**Affiliations:** 1 Division of Gastroenterology and Hepatology, Department of Medicine, Faculty of Medicine, Vajira Hospital, Navamindradhiraj University, 681 Samsen Road, Dusit District, Bangkok, 10300 Thailand; 2 Division of Gastroenterology, Phra Nakhon Si Ayutthaya Hospital, Ayutthaya, Thailand; 3 Division of Gastroenterology, Panyananthaphikkhu Chonprathan Medical Center, Nonthaburi, Thailand; 4 Division of Gastroenterology, Banphaeo General Hospital, Samut Sakhon, Thailand

**Keywords:** Acute upper gastrointestinal hemorrhage, Metoclopramide, Modified Avgerinos Score, Endoscopic mucosal visualization, Length of hospital stay, Gastroenterology, Medical research

## Abstract

Acute upper gastrointestinal hemorrhage (UGIH) is the most common emergency condition that requires rapid endoscopic treatment. This study aimed to evaluate the effects of pre-endoscopic intravenous metoclopramide on endoscopic mucosal visualization (EMV) in patients with acute UGIH. This was a multicenter, randomized, double-blind controlled trial of participants diagnosed with acute UGIH. All participants underwent esophagogastroduodenoscopy within 24 h. Participants were assigned to either the metoclopramide or placebo group. Modified Avgerinos scores were evaluated during endoscopy. In total, 284 out of 300 patients completed the per-protocol procedure. The mean age was 62.8 ± 14.3 years, and 67.6% were men. Metoclopramide group achieved a higher total EMV and gastric body EMV score than the other group (7.34 ± 1.1 vs 6.94 ± 1.6; P = 0.017 and 1.80 ± 0.4 vs 1.64 ± 0.6; P = 0.006, respectively). Success in identifying lesions was not different between the groups (96.5% in metoclopramide and 93.6% in placebo group; P = 0.26). In the metoclopramide group, those with active variceal bleeding compared with the control group demonstrated substantial improvements in gastric EMV (1.83 ± 0.4 vs 1.28 ± 0.8, P = 0.004), antral EMV (1.96 ± 0.2 vs 1.56 ± 0.6, P = 0.003), and total EMV score (7.48 ± 1.1 vs 6.2 ± 2.3, P = 0.02). Pre-endoscopic intravenous metoclopramide improved the quality of EMV in variceal etiologies of UGIH, which was especially prominent in those who had signs of active bleeding based on nasogastric tube assessment.

Trial Registration: Trial was registered in Clinical Trials: TCTR 20210708004 (08/07/2021).

## Introduction

Acute upper gastrointestinal hemorrhage (UGIH) is the most common emergency condition. In the majority of patients with UGIH, hemorrhage can resolve spontaneously. However, a minority of patients without specific endoscopic management have a high mortality rate. In Thailand, the 30-day mortality of non-variceal UGIH is about 4.6%^1^. The time to endoscopy after a visit to the hospital and good mucosal visualization are both important factors for successful management. In a previous study, although patients received standard endoscopy within 24 h, in patients with a blood clot in the fundus with poor visualization during endoscopy, gastric points of bleeding could not be detected causing an event of rebleeding in 54% of the patients, leading to longer period of hospitalization, more blood transfusion unit requirement, and higher chances of surgical treatment^2^.

Administering prokinetic agents before esophagogastroduodenoscopy (EGD) has an important role in treating patients with UGIH. Erythromycin is one of the antibiotics with a high safety profile; it increases gastric peristalsis and decreases gastric emptying time resulting in enhanced visualization of gastric mucosa during emergency endoscopy and reduces the length of hospitalization, rate of admission, rate of repeat endoscopy, and the need for blood transfusion^3^. Based on previous studies, pre-endoscopic use of erythromycin has no serious adverse events and is cost-effective^3–7^. According to expert review's recommendation for non-variceal UGIH, endoscopists may order a single dose of intravenous prokinetics with erythromycin in selected patients prior to endoscopy for improved gastric visualization, especially in the fundus^8,9^. The underuse of prokinetics for pre-endoscopic preparation in UGIH may decrease the opportunity for good visualization^10^.

Metoclopramide is one of the prokinetic drugs which acts as both dopamine-2 receptor antagonists and stimulates serotonin receptor agonists (5HT_4_) in the gastrointestinal system. It decreases gastric emptying time without increasing biliary and pancreatic mobility. Two published abstracts on pre-endoscopic administered metoclopramide in patients with UGIH, who presented with overt hematemesis or melena with or without gastric lavage, did not find metoclopramide to be superior to erythromycin or placebo; however, both studies had a limited sample size and reported no adverse events^11,12^. The recent randomized controlled trial reported the pre-endoscopic metoclopramide improved gastric visualization in those with active UGIB due to gastric lesions and the need for a second look EGD^13^. Currently, there is no consensus on efficacy of metoclopramide to be used in pre-endoscopic preparation for mucosal visualization in a large UGIH population. According to Thailand guidelines, pre-endoscopic intravenous erythromycin is not recommended due to unavailability. Our primary aim was to evaluate the effects of using pre-EGD intravenous metoclopramide in patients with active UGIH in comparison to placebo in terms of gastric endoscopic mucosal visualization (EMV). The secondary endpoint was an episode of repeat endoscopy, lesion identification, adverse events, and hospitalization.

## Material and methods

### Study population and pre-endoscopic management

This multicenter study was conducted on patients referred to several tertiary centers in Thailand from December 2020 to November 2022. These centers included Vajira Hospital, Navamindradhiraj University, Phra Nakhon Si Ayutthaya Hospital, Panyananthaphikkhu Chonprathan Medical Center, and Banphaeo General Hospital. A total of 300 patients aged ≥ 18 years with either previously diagnosed UGIH or with new UGIH diagnosed during admission, were considered eligible for inclusion. Any symptom such as melena, hematochezia, coffee-ground stomach contents, and hematemesis was considered as a symptom of active UGIH. All eligible patients with UGIH who gave informed consent were enrolled. Exclusion criteria were a history of psychosis, a high risk for metoclopramide-related side-effects (such as a history of movement disorder, Parkinson's disease, pregnancy or lactation, and chronic kidney disease of stage 4 or above), a history of metoclopramide allergy, a history of gastrointestinal surgery, and unwillingness to participate. In order to address the safety concerns associated with metoclopramide-related side effects, participants who experienced initial drowsiness or agitation were excluded from the study. Eligible participants received initial resuscitation according to a standard universal guideline of UGIH^8,10,14^. In the majority of participants, nasogastric tube insertion and lavage were performed to evaluate the severity of bleeding. Intravenous fluid resuscitation was provided, and participants were administered leucocyte-poor packed red cells (LPRC) at a liberal hemoglobin threshold (< 10 g/dL) or restrictive hemoglobin threshold (< 8 g/dL), according to the underlying comorbidity^15^. The initial cause of bleeding was assessed and intravenous continuous infusion of somatostatin and intravenous proton pump inhibitors were provided in those suspected of variceal hemorrhage and non-variceal hemorrhage, respectively.

All participants were eligible for early EGD within 24 h after the first diagnosis of UGIH irrespective of their referral to emergency room, outpatient, or inpatient setting. The diagnosis of UGIH was based on clinical symptoms including melena, hematochezia, and coffee-ground stomach contents. Modified Avgerinos Score was used for estimating mucosal visualization as the score has been validated and proven reliable in a previous study^16^. The endoscopist evaluated and scored the lesions according to the difficulty of the endoscopic field. A full stomach was defined if more than half of the gastric mucosa was filled by fresh blood/blood clots. Visibility points were classified as 0: < 25% visible; 1: 25%–75% visible; and 2: more than 75% visible. Each area of the stomach was scored with a total maximum score of 8. A clear stomach was represented with a score of ≥ 6 while a full stomach had a score of ≤ 5 (Table supplement 1).

For participants who were categorized as Forrest classifications I and II, an endoscopic therapeutic intervention was provided according to a standard guideline. We defined a high-risk ulcer bleeding group as any participants who had active spurting/oozing, non-bleeding visible vessel, or adherent clot. For post-EGD high-risk endoscopic patients, who had undergone successful endoscopic therapy, intravenous proton pump inhibitors were continuously administered for at least 72 h. Repeat or second-look endoscopy was required either after failure to identify a lesion in early endoscopy or if rebleeding occurred after the first endoscopy.

### Ethical approval

The study protocol was conducted according to the ethical guidelines of the 1975 Declaration of Helsinki and was approved by the Institutional Review Board Faculty of Medicine Vajira Hospital (COA186/63). After explanation of the detail of the research concept, all the participants provided informed consent before participation. The necessity of trial registration was not acknowledged until 20% of the participants had been enrolled, and thus the trial was registered at the Thai Clinical Trial Registry; registration number: TCTR 20210708004.

### Sample size calculation

The sample size was calculated according to the value of mean and standard deviation (SD) of the EMV score in the reference study with type I and type II error and pooled variance with the formula given below. We adjusted the sample size up to 5% of the calculated numbers of population. Accordingly, 100 participants were enrolled in each group.

$$n = \frac{{2(Z_{1 - \alpha /2} + Z_{\beta } )^{2} \sigma^{2} }}{{(\mu_{0} - \mu_{1} )^{2} }}$$

*n* = Sample size; $Z_{\alpha }$ = type I error, α = 5%; $Z_{\beta }$ = type II error, β = 20%, $\sigma^{2}$ = The variance for population data is denoted by SD^2^, $\mu_{0}$, $\mu_{1}$ = mean of EMV score of control and intervention group.

### Randomized strategy and intervention

All participants received standard treatment for UGIH. Eligible patients were randomly assigned to either intravenous metoclopramide or placebo groups by a block of four randomizations. Treatments were delivered in a double-blinded manner. In a prior study, the pre-operation intravenous dose of metoclopramide was 10–20 mg, administered at least 5 min before the procedure^17^. Both groups of participants were administered either intravenous metoclopramide 10 mg (2 mL) or placebo 2 mL, 10 to 30 min prior the endoscopy by an endoscopic nurse. Additionally, an endoscopic team is present to closely monitor any adverse events that may occur. Both the endoscopists and the participants were blinded during all the procedure. All endoscopy maneuvers and therapeutic techniques for stopping bleeding were applied to all the participants according to the standard of treatment for acute UGIH.

### Endoscopic assessment

**Figure 1:**
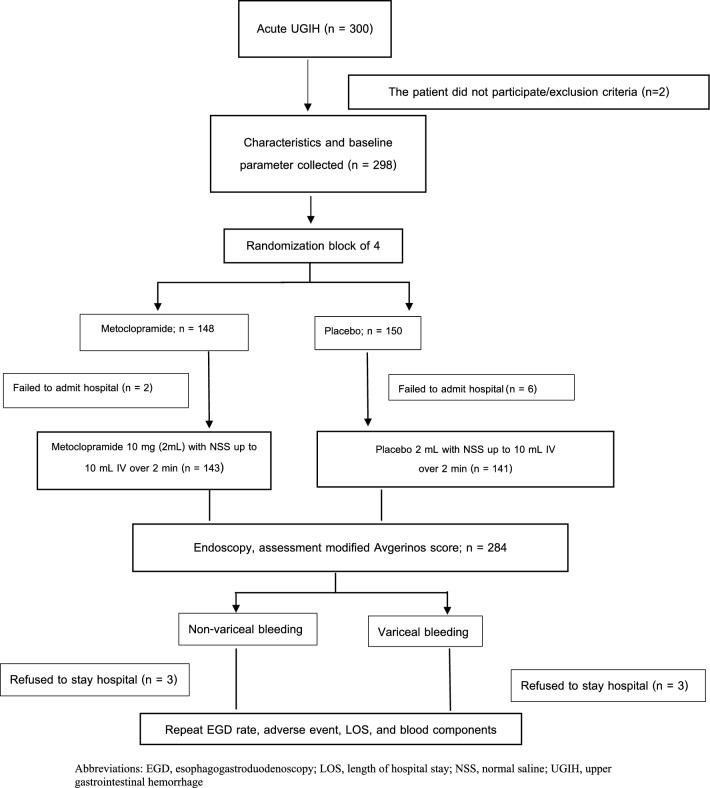
Flow chart of enrollment protocol. *EGD* esophagogastroduodenoscopy, *LOS* length of hospital stay, *NSS* normal saline, *UGIH* upper gastrointestinal hemorrhage.

All participants received early endoscopy within 24 h after diagnosis of UGIH. The endoscopic visualization used a modified Avgerinos Score^16^ assessment divided into 4 parts: fundus, body, antrum, and bulb. Each part was graded between 0–2 points. The score was assessed by one endoscopist from each of the four centers. All of them had undergone pre-endoscopic assessment training, were tested for the accuracy of visualization, and were qualified with more than 90% of the total test score. Pre-endoscopic Rockall, Blatchford, and AIM-65 scores were calculated. Endoscopic findings, endoscopic time, episode of repeat endoscopy, adverse event, length of hospital stay, and unit of required blood components were recorded. All participants were treated with standard UGIH treatment guidelines and were observed until discharge or death. Patients who denied admission for any reason or refused to stay after endoscopy were excluded from the analysis (Fig. 1).

### Statistical analysis

As previously described, the sample size was calculated using a formula that compares means from two independent samples. Patient characteristics and baseline parameters between groups were categorized as qualitative and quantitative data. The value is presented as mean ± SD and n (%). The P-value corresponded to Independent *t* test or Mann–Whitney U and Chi-square test. Endoscopic findings, duration, and comparison of repeat endoscopy requirements and adverse events are presented as n (%). The P-value corresponds to the Chi-square test. All analyses were performed by SPSS for Windows Version 27.0. Two-tailed P-values < 0.05 were considered statistically significant.

## Results

A total of 300 participants were diagnosed with acute UGIH. Two patients refused to participate. All remaining participants were randomly grouped into either the metoclopramide or control group. Eight participants achieved incomplete study protocol (6 in the placebo and 2 in the metoclopramide group). Six participants in the placebo group (n = 3) and metoclopramide group (n = 3) refused to stay in the hospital. A total of 284 patients who completed the process of care were eligible for analysis (Fig. 1). Baseline characteristics of participants in both groups were not different (Table 1). Approximately two-thirds of the participants (67.6%) were men. The mean age was 62.8 ± 14.3 years. Nearly one-third of the participants had a history of non-steroidal anti-inflammatory drug use (32.7%), followed by antiplatelet (25.7%), and anticoagulant (9.2%) use. Melena was the most predominant symptom presentation (55.63%), followed by hematochezia (9.15%). During nasogastric lavage, coffee-ground stomach contents were observed in 48.9% patients, fresh blood or blood clot in 31.33%, and food or bile content in the rest of the patients. The prevalence of melena was higher in the metoclopramide group than that in the placebo group. (63.6% vs 47.5%; P = 0.006). There was no difference between the two groups in prior medication for underlying disease and pre-endoscopic scoring evaluation including Rockall and AIM-65 score (2.28 ± 1.6 vs 2.18 ± 1.4; P = 0.57 and 1.47 ± 1.1 vs 1.37 ± 1.1; P = 0.45, for metoclopramide and placebo groups, respectively). All our participants had Glasgow Blatchford score of more than 1, which requires hospitalization or inpatient endoscopy. A trend of higher Glasgow-Blatchford score was seen in the metoclopramide group (9.24 ± 4.3 vs 8.27 ± 4.2; P = 0.054). The LPRC units transfused and the mean length of admission days was not different between the metoclopramide and placebo group (1.78 ± 2.2 vs 1.84 ± 1.9, P = 0.80; 5.8 vs 5.2; P = 0.49, respectively). Time from clinical diagnosis to endoscopy was not different between the metoclopramide and placebo group (16.96 ± 6.2 vs 17.93 ± 5.6 h; P = 0.16).

**Table 1:** Characteristics, pre-endoscopic evaluation of acute upper gastrointestinal bleeding.

	Non variceal (n = 233)			Variceal (n = 51)			Total (n = 284)		
	Metoclopramide (n = 118)	Placebo (n = 115)	*P*	Metoclopramide (n = 25)	Placebo (n = 26)	*P*	Metoclopramide (n = 143)	Placebo (n = 141)	*P*
Age (years)	63.66 ± 14.3	64.35 ± 14.7	0.719	56.08 ± 9.8	58.73 ± 13.4	0.428	62.34 ± 13.9	63.31 ± 14.6	0.566
Males	79 (66.9%)	75 (65.2%)	0.780	19 (76%)	19 (73.1%)	0.811	98 (68.5%)	94 (66.7%)	0.737
Comorbidities	101 (85.6%)	91 (79.1%)	0.195	20 (80%)	23 (88.5%)	0.406	121 (84.6%)	114 (80.9%)	0.401
Cirrhosis	22 (18.6%)	19 (16.5%)	0.671	16 (64%)	15 (57.7%)	0.645	38 (26.6%)	34 (24.1%)	0.634
Antiplatelet use	28 (23.7%)	38 (33%)	0.115	4 (16%)	3 (11.5%)	0.643	32 (22.4%)	41 (29.1%)	0.196
Anticoagulant use	15 (12.7%)	9 (7.8%)	0.220	0 (0%)	2 (7.7%)	0.157	15 (10.5%)	11 (7.8%)	0.432
NSAIDs use	43 (36.4%)	38 (33%)	0.586	5 (20%)	7 (26.9%)	0.560	48 (33.6%)	45 (31.9%)	0.767
Melena	77 (65.3%)	57 (49.6%)	0.015	14 (56%)	10 (38.5%)	0.210	91 (63.6%)	67 (47.5%)	0.006
Hematochezia	10 (8.5%)	10 (8.7%)	0.952	2 (8%)	4 (15.4%)	0.413	12 (8.4%)	14 (9.9%)	0.653
Systolic BP (mmHg)	121.1 ± 27.0	122.41 ± 26.3	0.710	113.44 ± 19.6	121.08 ± 17.7	0.152	119.76 ± 26.0	122.16 ± 24.9	0.429
Hypotension	67 (56.8%)	57 (49.6%)	0.270	8 (32%)	7 (26.9%)	0.691	75 (52.4%)	64 (45.4%)	0.234
Nasogastric lavage content			0.236			0.253			0.086
Coffee ground	61 (51.7%)	61 (53%)		8 (32%)	9 (34.6%)		69 (48.3%)	70 (49.6%)	
Fresh blood/Blood clot	30 (25.4%)	30 (26.1%)		13 (52%)	16 (61.5%)		43 (30.1%)	46 (32.6%)	
No content	8 (6.8%)	14 (12.2%)		0 (0%)	1 (3.8%)		8 (5.6%)	15 (10.6%)	
Food content	15 (12.7%)	5 (4.3%)		2 (8%)	0 (0%)		17 (11.9%)	5 (3.5%)	
Bile content	2 (1.7%)	3 (2.6%)		0 (0%)	0 (0%)		2 (1.4%)	3 (2.1%)	
Hemoglobin (g/dL)	8.38 ± 2.8	8.58 ± 2.9	0.599	7.81 ± 2.1	8.75 ± 2.8	0.188	8.28 ± 2.7	8.61 ± 2.9	0.325
Platelet × 10^3^ (cell/mm^3^)	234.5 ± 148.3	234.2 ± 107.0	0.986	144.1 ± 75.4	164.1 ± 87.7	0.388	218.7 ± 142.4	221.3 ± 107	0.863
Prothrombin time (s)	16.55 ± 15.8	16.03 ± 14.1	0.795	16.5 ± 3.1	19.5 ± 14.2	0.304	16.54 ± 14.4	16.67 ± 14.2	0.936
Glasgow Blatchford score	9.36 ± 4.3	8.38 ± 4.3	0.086	8.72 ± 4.4	7.77 ± 3.5	0.405	9.24 ± 4.3	8.27 ± 4.1	0.054
Pre-endoscopic Rockall score	2.23 ± 1.6	2.19 ± 1.4	0.855	2.52 ± 1.2	2.12 ± 1.2	0.254	2.28 ± 1.6	2.18 ± 1.4	0.568
AIM-65	1.45 ± 1.09	1.38 ± 1.08	0.640	1.56 ± 1.23	1.31 ± 1.19	0.46	1.47 ± 1.11	1.37 ± 1.1	0.448
Pre-endoscope LPRC unit(s)	1.81 ± 2.39	1.77 ± 1.83	0.886	1.68 ± 1.35	2.19 ± 2.1	0.303	1.78 ± 2.24	1.84 ± 1.88	0.805
Time from diagnosis of UGIH to EGD (h)	17.32 ± 6.07	18.29 ± 5.43	0.303	15.24 ± 7.06	17.19 ± 6.47	0.309	16.96 ± 6.28	17.93 ± 5.62	0.169

*BP* blood pressure, *LPRC* leukocyte poor red cell, *NSAIDs* non-steroidal anti-inflammatory drugs, *UGIH* upper gastrointestinal hemorrhage, *EGD* esophagogastroduodenoscopy.

All participants underwent EGD within 24 h after diagnosis. Time from diagnosis of UGIH to EGD was not different between variceal and non-variceal groups (16.14 ± 6.4 vs. 18.09 ± 7.2 h; P = 0.09). The mean duration of endoscopy was not different between the metoclopramide (18.12 ± 13 min) and placebo groups (17.43 ± 15.6 min; P = 0.70; Table 2). The majority of participants (82.04%) were found to have an etiology of non-variceal bleeding. In the metoclopramide group, total EMV was significantly higher than that in the placebo group (7.34 ± 1.1 vs 6.94 ± 1.6; P = 0.017). The average visualized score at the body was also superior in the metoclopramide group (1.80 ± 0.4 vs 1.64 ± 0.6; P = 0.006). There was no significant difference between the two groups in respect of EMV in other gastric areas beyond the gastric body. However, the superiority of pre-endoscopic metoclopramide over placebo in improving total, body, and antral EMV was observed in only variceal subgroup (total EMV: 7.52 ± 1.0 vs 6.27 ± 2.3, P = 0.017; body EMV: 1.84 ± 0.3 vs 1.31 ± 0.7, P = 0.004; antral EMV: 1.96 ± 0.2 vs 1.58 ± 0.5, P = 0.003; Table 3).

**Table 2:** Endoscopic findings and etiology of gastrointestinal bleeding.

	Metoclopramide (n = 143)	Placebo (n = 141)	P
Time from diagnosis of UGIH to EGD (h)	16.96 ± 6.28	17.93 ± 5.62	0.169
Duration of endoscopy (min)	18.1 ± 13.97	17.43 ± 15.58	0.705
Etiology of bleeding			
Variceal bleeding			
Esophageal varices	23 (16.1%)	19 (13.5%)	0.536
Gastric varices	2 (1.4%)	7 (5%)	0.086
Non-variceal bleeding	118 (82.5%)	115 (81.6%)	0.834
Mallory Weiss tear	10 (7%)	16 (11.3%)	0.203
Portal hypertensive gastropathy	6 (4.2%)	5 (3.5%)	0.777
Gastritis	6 (4.2%)	5 (3.5%)	0.777
Peptic ulcer	96 (67.1%)	89 (63.1%)	0.478
Forrest classification of ulcer			0.118
Ia (Spurting hemorrhage)	0 (0%)	1 (1.1%)	
Ib (Oozing hemorrhage)	6 (6.3%)	5 (5.6%)	
IIa (Non-bleeding visible vessel)	20 (20.8%)	8 (9%)	
IIb (Adherent clot)	1 (1%)	5 (5.6%)	
IIc (Flat pigmented spot)	16 (16.7%)	18 (20.2%)	
III (Clean ulcer base)	53 (55.2%)	52 (58.4%)	
Endoscopic intervention	49 (34.3%)	47 (33%)	0.868

*UGIH* upper gastrointestinal hemorrhage, *EGD* esophagogastroduodenoscopy.

**Table 3:** Endoscopic mucosal visualization of upper gastrointestinal bleeding.

	Non variceal (n = 233)			Variceal (n = 51)			Total (n = 284)		
	Metoclopramide (n = 118)	Placebo (n = 115)	P	Metoclopramide (n = 25)	Placebo (n = 26)	P	Metoclopramide (n = 143)	Placebo (n = 141)	P
Endoscopic visualization score									
Fundus	1.85 ± 0.38	1.76 ± 0.54	0.141	1.76 ± 0.52	1.54 ± 0.76	0.230	1.83 ± 0.41	1.72 ± 0.59	0.056
Body	1.8 ± 0.44	1.71 ± 0.47	0.166	1.84 ± 0.37	1.31 ± 0.79	0.004*	1.8 ± 0.43	1.64 ± 0.56	0.006*
Antrum	1.79 ± 0.45	1.76 ± 0.51	0.615	1.96 ± 0.2	1.58 ± 0.58	0.003*	1.82 ± 0.42	1.72 ± 0.52	0.094
Bulb	1.87 ± 0.33	1.87 ± 0.36	0.942	1.96 ± 0.2	1.88 ± 0.43	0.430	1.89 ± 0.32	1.87 ± 0.38	0.702
Total EMV	7.31 ± 1.16	7.1 ± 1.39	0.212	7.52 ± 1.05	6.27 ± 2.31	0.017*	7.34 ± 1.14	6.94 ± 1.62	0.017*

*EMV* endoscopic mucosal visualization.

*Statistical significance.

**Figure 2:**
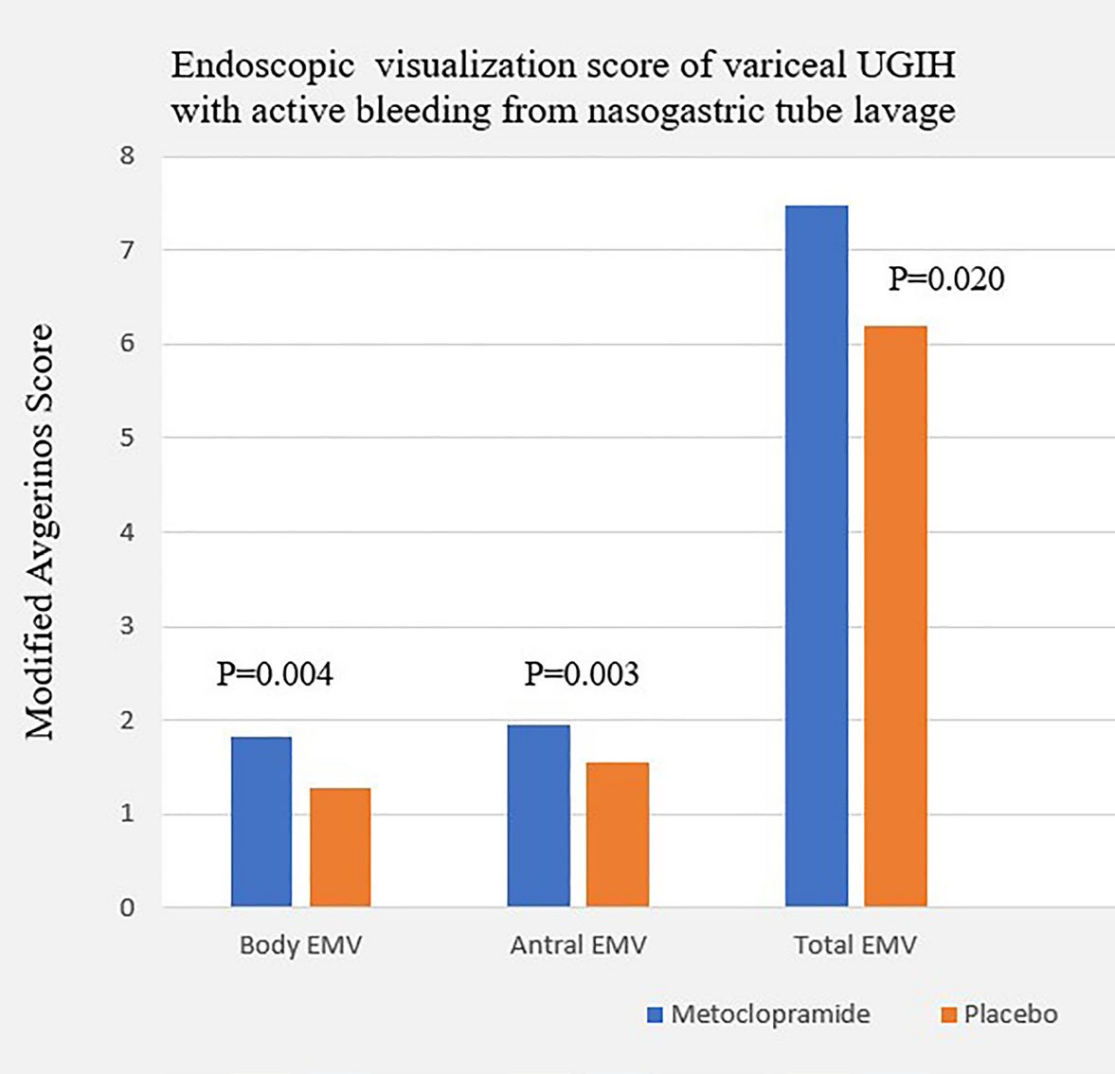
Endoscopic visualization score of variceal UGIH with active bleeding from nasogastric tube lavage. *UGIH* upper gastrointestinal hemorrhage, *EMV* endoscopic mucosal visualization.

In the first attempt of EGD, the cause of UGIH was identified in 96.5% and 93.6% of the participants in the metoclopramide and placebo groups, respectively (P = 0.26). More than half of the individuals (65.14%) had an etiology of peptic ulcer bleeding (67.1% in metoclopramide and 63.1% in placebo group), which was followed by variceal hemorrhage (17.95%), Mallory Weiss tears (9.15%), bleeding portal gastropathy (3.87%), and gastritis (3.87%). In patients with ulcer bleedings (185 patients), the location of peptic ulcers was found at the antrum (49.18%), duodenum (43.7%), pylorus (17.29%), body (9.18%), and fundus (5.4%). Among those with peptic ulcer bleeding, less than 10% of patients had active spurting or oozing (Forrest Ia and Ib), and more than one-third (36.97%) of all patients with UGIH had a clean-based ulcer (Forrest III). In 46 participants with high-risk ulcer, the majority of lesions were found at the duodenum with a non-significant difference in either the total EMV score or the volume of water irrigation between the metoclopramide and placebo groups (7.30 ± 0.9 vs. 6.68 ± 1.8, P = 0.15; 82.96 vs. 109.47 mL, P = 0.50; Table 4). Interestingly, patients in the metoclopramide group had greater body and total EMV visualization scores than those in the placebo group in 234 patients with UGIH with fresh blood, blood clot, or coffee-ground stomach contents (1.78 ± 0.5 vs 1.59 ± 0.6, P = 0.006; 7.28 ± 1.2 vs 6.8 ± 1.7; P = 0.017). The enhanced total, body, and antral visualization was obviously seen only in the variceal group who had signs of active bleeding based on nasogastric tube assessment (total EMV: 7.48 ± 1.1 vs 6.2 ± 2.3, P = 0.02; body EMV: 1.83 ± 0.4 vs 1.28 ± 0.8, P = 0.004; antral EMV: 1.96 ± 0.2 vs 1.56 ± 0.6, P = 0.003; Table 5*, *Fig. 2).

**Table 4:** Endoscopic high- risk ulcer categorized by Forrest classification *

	Metoclopramide (n = 27)	Placebo (n = 19)	P
Duration of endoscopy (min)	32.11 ± 21.99	28.58 ± 20.91	0.587
Endoscopic visualization score			
Fundus	1.96 ± 0.19	1.63 ± 0.68	0.053
Body	1.85 ± 0.36	1.68 ± 0.58	0.275
Antrum	1.81 ± 0.4	1.79 ± 0.54	0.854
Bulb	1.67 ± 0.48	1.58 ± 0.61	0.587
Total score	7.3 ± 0.99	6.68 ± 1.83	0.150
Lesion identification position			
First part duodenum	20 (74.1%)	9 (47.4%)	0.065
Second part duodenum	12 (44.4%)	5 (26.3%)	0.210
Antrum	11 (40.7%)	6 (31.6%)	0.526
Pylorus	5 (18.5%)	3 (15.8%)	0.810
Body	0 (0%)	1 (5.3%)	0.228
Fundus	0 (0%)	1 (5.3%)	0.228
Volume of water (mL)	82.96 ± 128.2	109.47 ± 134.27	0.502

*High- risk ulcer categorized by Forrest classification Ia (Spurting hemorrhage), Ib (Oozing hemorrhage), IIa (Non-bleeding visible vessel) and IIb (Adherent clot).

**Table 5:** Endoscopic visualization score of non-variceal vs variceal bleeding in the subgroup with fresh blood/blood clot or coffee ground.

	Coffee ground and fresh blood/blood clot								
	Non variceal (n = 186)			Variceal (n = 48)			Total (n = 234)		
	Metoclopramide (n = 93)	Placebo (n = 93)	P	Metoclopramide (n = 23)	Placebo (n = 25)	P	Metoclopramide (n = 116)	Placebo (n = 118)	P
Fundus	1.83 ± 0.41	1.72 ± 0.56	0.135	1.74 ± 0.54	1.52 ± 0.77	0.264	1.81 ± 0.44	1.68 ± 0.61	0.058
Body	1.77 ± 0.47	1.68 ± 0.49	0.172	1.83 ± 0.39	1.28 ± 0.79	0.004*	1.78 ± 0.45	1.59 ± 0.59	0.006*
Antrum	1.76 ± 0.48	1.72 ± 0.54	0.565	1.96 ± 0.21	1.56 ± 0.58	0.003*	1.8 ± 0.44	1.69 ± 0.55	0.078
Bulb	1.86 ± 0.35	1.84 ± 0.4	0.696	1.96 ± 0.21	1.88 ± 0.44	0.452	1.88 ± 0.33	1.85 ± 0.41	0.510
Total EMV	7.23 ± 1.21	6.96 ± 1.46	0.173	7.48 ± 1.08	6.2 ± 2.33	0.020*	7.28 ± 1.18	6.8 ± 1.7	0.013*

*EMV* endoscopic mucosal visualization.

There was no difference in the rate of re-EGD between the metoclopramide and placebo groups (3.5% vs 4.3%; P = 0.74). After early rescue EGD, 11 of 14 participants (78.57%) underwent a second-look endoscopy due to failure of the first-EGD in lesion identification in 3 participants (gastric ulcer in one patient, portal hypertensive gastropathy in one patient, and duodenal ulcer in one patient), rebleeding in 4 participants (gastric ulcer in three patients and esophageal varices in one patient), and follow up endoscopy after first therapeutic intervention of gastric ulcer bleeding (in four patients). After a single dose of metoclopramide, no adverse event was observed. Fourteen participants did not survive (9 participants [6.4%] in the placebo and 5 [3.5%] in the metoclopramide group; P = 0.26). Three participants (1.05%) died from uncontrolled bleeding, while the others died from pre-admission sepsis (four patients), malignancy (two patients), ruptured aortic aneurysm (one patient), myocardial infarction (two patients), decompensated liver disease (one patient), and chronic obstructive pulmonary disease with acute exacerbation (one patient). Two participants in the placebo group had post-endoscopy aspiration pneumonia.

## Discussion

Upper gastrointestinal bleeding is an emergency condition that requires rapid endoscopic management. A barrier to mucosal visualization in UGIH is active bleeding, which may obscure the lesion identification performance of the endoscopists^2^. Metoclopramide is a prokinetic drug that affects both dopamine 2 receptor antagonists and serotonin (5HT4) receptor agonists and accelerates both gastric contraction and gastric emptying time by augmenting acetylcholine release from enteric neurons and stimulating muscarinic receptor smooth muscles of the gastrointestinal system^1,2,4,5,18^. Previous studies by Sussman et al. compared administration of intravenous metoclopramide with a placebo in patients with overt hematemesis/melena without prior gastric lavage. A non-significant trend in the improvement of EMV score was noted even with a longer gap between metoclopramide administration and endoscopy compared to that in our study; however, the small number of participants was a limitation of the previous study^12^. Further, a meta-analysis of prokinetic agents was performed on a small population and a majority of the participants had clinical hematemesis/fresh blood, and compared with placebo, use of prokinetic agents showed a reduction in the need for the second round of EGD; however, endoscopic visualization was not reported because of no consensus in the definition of visualization^3^. The previous abstract provided evidence of the efficacy of metoclopramide in treating EMV in a limited sample size. However, it did not specifically investigate the impact on active fresh blood or the etiologic subgroup^19^. Therefore, we decided to collect a study on large population of UGIH from multiple centers to evaluate the effect of metoclopramide in mucosal visualization by calibrating the grading points of visualization among all endoscopists using the Modified Avgerinos score.

Even though the recent study included participants with hematemesis or the presence of fresh blood in the nasogastric tube, the limited sample size resulted in a non-significant trend of a higher proportion of patients with adequate visualization as indicated by visualization scores in the metoclopramide group^13^. Our study shows the benefit of intravenous metoclopramide in enhancement of mucosal visualization by demonstrating not only an increase in total EMV but also in gastric body visualization scores with heterogeneity in the clinical presentation of UGIH. Despite the average difference in EMV scores between the groups was minimal, it is noteworthy that the metoclopramide group had a statistically significant advantage in terms of mucosal visualization. We have identified a larger number of UGIB patients who have shown that metoclopramide is effective in improving visibility in UGIB cases with active bleeding in the nasogastric tube, particularly in cases of extremely active fresh blood in variceal etiology. Currently, when variceal bleeding is diagnosed, pre-endoscopic somatostatin is recommended^20–22^. However, the immediate effect of somatostatin is a reduction in portal pressure and blood flow, which is followed by a rapid rebound of hemodynamic status toward baseline. This study is the first to demonstrate the benefit of metoclopramide in EMV of patients with variceal bleeding and active fresh blood. We emphasized that pre-endoscopic metoclopramide, in addition to pre-endoscopic somatostatin infusion, may improve gastric blood pool in a subset of patients with esophageal variceal bleeding. Within our non-variceal participants, not only pure active fresh blood but also a substantial fraction of coffee grounds were examined. This research revealed a decrease in the effect of metoclopramide, resulting in a non-significant increase in EMV within this particular group.

In a previous randomized controlled trial (RCT) of a small number of participants, a large pre-endoscopic volume of gastric lavage significantly improved fundal EMV compared with EGD alone^23^. However, there are no large RCT trials for supporting pre-endoscopic gastric lavage^24^. According to a multicenter RCT trial pre-endoscopic gastric lavage with prokinetic drugs cannot improve EMV during endoscopy compared with endoscopy with prokinetic agents alone^25^. Adjunct to the use of pre-endoscopic non-invasive score assessment, we used a small volume of NSS gastric lavage, which had less impact on EMV, to estimate the severity of active bleeding.

A standard pre-endoscopic preparation along with early endoscopy in all participants led to a high success rate of lesion identification in our study, which is comparable with previous reports^26^. The proportion of identified lesions was non-statistically significant between metoclopramide and placebo groups due to multiple reasons: first, more than half of our patients presented with melena, which was bound to reveal less blood content in the proximal stomach as compared to fresh blood/hematemesis, and second, apart from the use of pre-endoscopic prokinetic agents, identification of lesions may depend on the expertise of endoscopists including the endoscopic techniques. Metoclopramide is unable to reduce the duration of endoscopy due to this particular reason. Despite a better visualization quality, a non-significant decrease in the endoscopic time was also observed in our variceal hemorrhage study group. To further clarify this, we believe that there is a need to perform future studies with higher number of participants with variceal bleeding.

We had a minority of participants who were categorized as high-risk for ulcer bleeding (classified as Forrest Ia, Ib, IIa, and IIb), and no significant difference in EMV score was found in this group for two reasons. First, more than half of the ulcers were identified in the duodenum, which is not affected by the EMV enhancement by the pharmacologic action of metoclopramide. Second, only a few participants in our high-risk ulcer bleeding group had identified lesions within the fundus or body. In the future, further studies to evaluate the supporting benefit of pre-EGD metoclopramide in body visualization or clinical outcomes impact should be conducted by enrolling more participants with non-variceal bleeding and high-risk Forrest classification.

Among patients with the comparable pre-endoscopic score, times of endoscopy requirement, and lesion identification and therapeutic intervention rate between metoclopramide and placebo groups, this study did not demonstrate the difference in pre- and post-endoscopy LPRC unit transfusion, rate of repeat endoscopy, or length of admission stays in both groups.

Regarding the safety consideration associated with chronic oral metoclopramide use, short-term adverse effects including dystonic reaction, muscle spasm, agitation, dizziness and tardive dyskinesia should be taken into account. Previous research publications provide evidence that intravenous metoclopramide is safe for patients with active upper gastrointestinal hemorrhage; neither exacerbation of this condition nor severe adverse events were reported^12,13^. Our research utilized a single intravenous dose of metoclopramide, and there have been no documented adverse effects.

This study had several limitations. First, heterogenous clinical presentations in terms of active bleeding were included in our study. This factor caused a high diversity in the UGIB population. Second, we did not record fasting time which may affect the amount of blood content in stomach. Last, considering that the majority of our patients were diagnosed with non-high-risk ulcer and lesions requiring intervention were mostly distributed in the duodenum, IV metoclopramide did not show a substantial efficacy for them.

We believe that future studies should enroll a homogenous large population with variceal UGIH subgroup with active fresh blood and/or hematemesis regardless of pre-endoscopic nasogastric lavage for studying the benefits of pre-endoscopic metoclopramide administrations.

## Conclusion

Pre-endoscopic intravenous metoclopramide enhances the quality of EMV in patients with variceal etiology of UGIH. The drug may be a good alternative option for pre-endoscopic UGIH preparation.

### Supplementary Information

Supplementary Table 1.

## Data Availability

Data are available on reasonable request at supatsri@nmu.ac.th.
