# Supplementary material for: Intravenous metoclopramide for increasing endoscopic mucosal visualization in patients with acute upper gastrointestinal bleeding: a multicenter, randomized, double-blind, controlled trial
Source: Sci Rep. 2024 Mar 31;14:7598. doi: 10.1038/s41598-024-57913-2 (PMC10982284; doi:10.1038/s41598-024-57913-2)
Supplement: Supplementary file 1 — Supplementary Table 1. [file 41598_2024_57913_MOESM1_ESM.docx]

Table Supplement 1: Modified Avgerinos score

| **Visualization**  **Gastric Area** | **< 25%** | **25% – 75%** | **>75%** |
| --- | --- | --- | --- |
| **Fundus** | 0 | 1 | 2 |
| **Body** | 0 | 1 | 2 |
| **Antrum** | 0 | 1 | 2 |
| **Bulb** | 0 | 1 | 2 |
